# Coping with systemic lupus erythematosus in patients’ words

**DOI:** 10.1136/lupus-2022-000656

**Published:** 2022-05-12

**Authors:** Alain Cornet, Davide Mazzoni, Angela Edwards, Dario Monzani, Gabriella Pravettoni, Jeanette Andersen, Marta Mosca

**Affiliations:** 1 Lupus Europe, Brussels, Belgium; 2 Department of Oncology and Hemato-Oncology, University of Milan, Milano, Italy; 3 Applied Research Division for Cognitive and Psychological Science, IEO, European Institute of Oncology IRCCS, Milan, Italy; 4 Rheumatology Unit, Department of Internal Medicine, University of Pisa, Pisa, Italy

**Keywords:** psychology, qualitative research, quality of life

## Abstract

**Objective:**

Previous research on coping strategies of patients with SLE showed that there are no absolute adaptive or maladaptive strategies and that the range of potential coping strategies is large and heterogeneous. In this paper, we aimed to identify, in a large sample of patients with SLE (N=3222), the most frequent words used by patients to describe their coping strategies, to group them into significant themes and to test their possible association with specific patient characteristics.

**Methods:**

Our analyses were based on the data set of the European survey ‘Living with Lupus in 2020’ (N=3222). Through the T-LAB software, we analysed the answers that adult participants gave to an open-ended question about how they cope with the disease. We identified the most frequent words, and with hierarchical cluster analysis we grouped them into semantic clusters (ie, themes) that were characterised by specific patterns of words. Finally, we tested the possible association between clusters and illustrative variables (sociodemographics, disease characteristics, quality of life).

**Results:**

Five coping strategies were identified, each of them constituting an important percentage of the total word occurrences: positive attitude (22.58%), social support (25.46%), medical treatments (10.77%), healthy habits (20.74%) and avoid stress (20.45%). Each strategy was statistically associated with specific patient characteristics, such as age and organ involvement.

**Conclusions:**

Learning to adapt to a lifetime of having SLE may require replacing old coping strategies with more effective ones. Investigating patients’ coping strategies in relation to different patient characteristics represents a useful starting point for developing more targeted and efficacious interventions.

**Key messages**

**What is already known on this topic**

- The range of potential strategies to cope with SLE is large and heterogeneous.

**What this study adds**

- Based on 3222 cases, we identified five coping strategies: positive attitude (22.58%), social support (25.46%), medical treatments (10.77%), healthy habits (20.74%) and avoid stress (20.45%).

**How this study might affect research, practice and/or policy**

- Patients with certain characteristics (both demographics and disease-related) may be willing to use some specific coping strategies, more or less functional, and this could orient future research and interventions.

## Introduction

SLE is a chronic systemic autoimmune disease that can affect many organs, including the skin, lung, kidney, heart, brain and joints, with different grades of severity. Even if some clusters of clinical and immunological features have been identified, SLE exhibits considerable variation in its manifestations between individuals.^1 2^ SLE is much more frequent among women (about 90% of total diagnoses) than men and prevalence rates generally range from 20 to 70 per 100 000 persons, with great variability between studies.^3 4^

SLE generally involves a variety of severe symptoms, with periods of intense flares and periods of remission.^5–7^ The clinical picture of SLE is very complex as it is not only related to disease activity but also to damage accrual, comorbidities and sometimes drug toxicity. As a result of the disease and its treatment, there are several limitations imposed on patients’ job, leisure and social activities, leading to poorer quality of life among patients than the general population.^8–10^

Thanks to recent improvements in the treatment of SLE, in the last decades fewer patients are dying early in their disease, and the study of psychosocial factors that may increase the chance of an adequate quality of life in patients with SLE is gradually growing in importance.^11–14^ In this regard, several authors underline the potential benefits of engaging patients in the active management of their condition.^15 16^ The present work specifically focuses on the resources and strategies that patients with SLE report when they describe how they face their condition. Before presenting our results and their implication for interventions, in the next paragraph we briefly review previous psychosocial research on coping strategies and some of the recent studies that considered the coping strategies of patients with SLE.

### Coping with SLE

The literature offers a number of classifications of patients’ coping strategies, which are more or less stressor-dependent or generalisable to different situations. For example, a classic model distinguishes between approach and avoidance coping strategies.^17^ In an approach-oriented coping strategy, an individual attempts to deal with life events by actively approaching the stressor, for instance by seeking information and social support, by planning ahead and by attempting to solve the problems caused by the stressor. The opposite of the approach orientation is a movement away from the stressor, referred to as an avoidance dimension, which may consist, for example, of a disengaged way of relating to stressful events, diversion and escape.^18^

In another classic model of coping strategies, Lazarus and Folkman^19^ proposed the distinction between ‘emotion-focused’ coping and ‘problem-focused’ coping. Emotion-focused coping is usually defined as aiming to manage the emotional distress that is associated with the situation. Problem-focused coping refers to the actions to modify the problem at hand and typically includes elements such as generating options to solve the problem, evaluating the pros and cons of different options, and implementing steps to solve the problem.^19^

However, in the literature, the distinction between these dimensions (approach-avoidance and problem-emotion) is not always clear and they have been sometimes considered as overlapping and for their possible intersections.^20–23^ In this way, a number of coping strategies were identified, such as (to mention some examples) denial, self-blaming, avoidance, resignation, self-distraction, behavioural disengagement, active coping (ie, taking active steps to circumvent the stressor), planning, seeking instrumental and emotional social support, humour, venting, turning to religion, and use of substances.^20 23^

A recent line of research paid particular attention to the coping strategies that are adopted by patients with SLE to face the disease and the related stress.^24 25^ For example, a qualitative study with 13 adults taking part in focus groups identified some ‘modifiable factors’ (social support, open communication about SLE and strong patient–provider relationships) and some ‘strategies’ (focusing on the positive side of SLE, reducing stress and technology) that can be helpful to face the disease.^25^ In general, the literature suggests that an active coping style seems to help preserve patients’ quality of life.^24^ However, there are no absolute ‘adaptive’ or ‘maladaptive’ strategies, and the suitability of these depends on the situation that a patient could sustain.^13 26^ Moreover, the range of potential coping strategies is so large and heterogeneous that further efforts are required to identify the coping strategies that are more frequently associated with specific characteristics of the patient and the disease.

Taking advantage of the availability of the data set ‘Living with Lupus in 2020’, the aim of this study was to analyse the answers that adult patients with SLE gave to an open-ended question about the strategies that they used to cope with the disease, identifying the main themes.

## Methods

### Procedure and participants

Lupus Europe is the European umbrella organisation that brings together national lupus patient organisations from across Europe. Covering many European countries, it represents over 30 000 patients in their respective memberships. The Lupus Europe board is composed of six trustees, who are all either lupus patients themselves or relatives of a patient with lupus. In 2020, Lupus Europe launched an internal online survey named ‘Living with Lupus in 2020’. The main aim of the survey was to get a picture of the impact of the disease on patients with lupus in Europe in 2020. Participants were invited to take part in the survey through an invitation that was published on the website of Lupus Europe and was distributed also by the national lupus patient organisations.

The survey was anonymous and covered several areas, including information about sociodemographics, disease characteristics and patients’ quality of life. It was made available to European patients with lupus both through a unique link to a multilingual start page on Lupus Europe’s website or through national language-specific direct access links. Data were collected in the platform SurveyLegend and were then downloaded as an Excel file. More information about data collection and descriptive statistics on the entire sample are available in the publication by Cornet and colleagues.^7^

For the purposes of this study, all cases that reported missing values in one or more of the key variables were removed, as well as a minority of participants not reporting an SLE diagnosis. The final sample thus consisted of 3222 valid cases.

### Questionnaire

The focus of our work was on the *coping strategies* that were reported by patients. Participants were asked to identify the most relevant words to describe their ways of coping with their condition. The main variable (string) consisted of the answer to the question ‘What is the most important thing that helps you manage your condition?’ Patients were required to give a one-line textual answer (no more than 10 words) in their own language, which was subsequently translated to English for analyses.

Many *patient characteristics* were also assessed for their possible association with different coping strategies. Table 1 presents all the descriptive statistics. These variables included sociodemographics (gender, age, education and employment status), disease characteristics (years from the diagnosis, organ involvement and disease under control), quality of life (functional autonomy, perceived pain and discomfort, anxiety, and depression).

**Table 1:** Descriptive analyses of the illustrative variables

Variables	Variable level	Frequency (%)
Sociodemographic variables		
Gender	Male	119 (3.69)
	Female	3103 (96.31)
Age	18–40	1232 (38.24)
	41–60	1661 (51.55)
	61–87	329 (10.21)
Education	Up to secondary education	1736 (53.88)
	Higher education	1486 (46.12)
Employment status	Not working	1292 (40.10)
	Part-time working	645 (20.02)
	Full-time working	1285 (39.88)
Years from diagnosis	0–5	970 (30.11)
	6–15	1158 (35.94)
	16–70	1094 (33.95)
Organ involvement		
Skin	No	1307 (40.56)
	Yes	1915 (59.44)
Heart	No	2674 (82.99)
	Yes	548 (17.01)
Bloodstream	No	2343 (72.72)
	Yes	879 (27.28)
Lungs	No	2646 (82.12)
	Yes	576 (17.88)
Central nervous system	No	2683 (83.27)
	Yes	539 (16.73)
Muscles	No	1811 (56.21)
	Yes	1411 (43.79)
Joints	No	542 (16.82)
	Yes	2680 (83.18)
Kidneys	No	2247 (69.74)
	Yes	975 (30.26)
Antiphospholipid syndrome	No	2526 (78.40)
	Yes	696 (21.60)
Disease under control	No	908 (28.18)
	Yes	2314 (71.82)
Quality of life		
Functional autonomy	Low	1750 (54.31)
	High	1472 (45.69)
Pain and discomfort	Low	2005 (62.23)
	High	1217 (37.77)
Anxiety and depression	Low	2145 (66.57)
	High	1077 (33.43)

In regard to the quality of life dimensions, functional autonomy was assessed with three items about mobility (‘How do you assess your mobility, that is, your ability to walk around?’), self-care (‘How do you assess your ability to perform self-care tasks like washing or dressing yourself?’) and daily activity (‘How do you assess your ability to perform normal daily activities, like studying, working, housework, leisure or participation in family life’), with possible answers ranging from 1 ‘not able’ to 5 ‘no problem at all’.^7^ A Cronbach’s α value of 0.84 was acceptable and a mean index was calculated. Perceived pain/discomfort (‘How do you assess your level of discomfort or pain?’, with answers ranging from 1 ‘no pain/discomfort’ to 5 ‘extreme pain/discomfort’) and anxiety/depression (‘Do you feel anxious or depressed?’, with answers ranging from 1 ‘not at all’ to 5 ‘yes, extremely’) were assessed through single items.

### Analysis

The answers to the open-ended question about coping were analysed through the software T-LAB Plus 2021. This software represents a set of linguistic and statistical tools for content analysis and text mining.^27–29^ Consistent with our main aim, the analytical process followed three main steps.

1. Identification of the most frequent words used in patients’ answers. After the importation process (ie, a series of processes that transform the texts into a set of tables integrated in the T-LAB database), T-LAB allowed identification of the most frequent words that were used by the participants.
2. Identification of meaningful semantic clusters that are characterised by a specific pattern of words (ie, themes). We performed a singular value decomposition (SVD), followed by hierarchical cluster analysis. SVD is a technique for dimensionality reduction which can be used to discover the latent dimensions which determine semantic similarities between words (ie, the data table was a co-occurrence matrix whose rows and columns were keywords). Cluster analysis uses the results of the SVD to identify semantic clusters (ie, themes) that are characterised by a specific pattern of words. In this sense, our approach was ‘bottom-up’, that is, an inductive approach in which the themes identified are strongly linked to the data themselves.^27–30^
3. Testing of the possible association between clusters and specific respondent characteristics (ie, illustrative/categorical variables in the T-LAB environment). We tested the possible association between clusters and categorical variables through a *test value*,^31^ with a threshold value (test value >1.96) corresponding to statistical significance (p<0.05).

## Results

The mean age of the participants was 44.56 years (SD=12.24), with 3103 (96.31%) women and 119 (3.69%) men. The mean time from diagnosis (in years) was 12.82 (SD=10.20). In T-LAB, illustrative variables must be categorical, and for this reason the scales about quality of life were also dichotomised. The responses to the items about pain/discomfort, anxiety/depression and functional autonomy were provided on a range from 1 to 5. The items about pain/discomfort and anxiety/depression were recoded into ‘low’ (1–3) and ‘high’ (4–5). The value of functional autonomy was also recoded into ‘low’ (1–2) and ‘high’ (3–5). Table 1 presents all the descriptive statistics and how the scales were categorised to be used as categorical (illustrative) variables in T-LAB.

### Most frequent words

Table 2 presents the 30 most frequently reported words, including a wide range of actions, contexts and objects.

**Table 2:** Most frequently reported words

Lemma	Frequency	Lemma	Frequency	Lemma	Frequency
Family	445	Good	105	Walk	62
Rest	287	Husband	96	Pain	58
Medication	252	Life	94	Activity	57
Support	192	Medicine	94	Treatment	55
Positive	163	Think	89	Diet	48
Child	124	Work	88	Daughter	47
Exercise	120	Love	73	Regular	47
Friend	119	Attitude	73	Pace	47
Sleep	114	Sport	65	Understanding	47
Stress	111	Doctor	63	Hydroxychloroquine	46

As a second output of analysis, we obtained a five-cluster solution, which is presented in table 3. The table reports the word occurrences in each cluster and the list of keywords that are characteristics of the cluster (also see online supplemental file 1). Finally, at the bottom of table 3, all the illustrative variables that are significantly associated with each cluster are also reported.

10.1136/lupus-2022-000656.supp1 Supplementary data

**Table 3:** The five clusters

	Cluster 1: ’positive attitude’	Cluster 2: ’social support’	Cluster 3: ’trust in medical treatments’	Cluster 4: ‘healthy habits’	Cluster 5: ‘to avoid stress’
Total occurrence (%)	1434 (22.58)	1617 (25.46)	684 (10.77)	1317 (20.74)	1299 (20.45)
Words (occurrence)	Positive (163)	Family (445)	Good (105)	Exercise (120)	Rest (287)
	Child (124)	Support (192)	Treatment (55)	Work (88)	Medication (252)
	Life (94)	Friend (119)	Medical (43)	Sport (65)	Sleep (114)
	Think (89)	Husband (96)	Faith (34)	Walk (62)	Stress (111)
	Attitude (73)	Medicine (94)	Care (34)	Diet (48)	Regular (47)
	Pain (58)	Love (73)	Drug (32)	Dog (34)	Pace (47)
	Daughter (47)	Doctor (63)	Listen (27)	Healthy (34)	Hydroxychloroquine (46)
	Help (45)	Activity (57)	Therapy (25)	Movement (31)	Yoga (35)
	Disease (45)	Understanding (47)	Know (22)	Eat (29)	Avoid (33)
	Live (44)	Positivity (42)	Follow-up (19)	Active (27)	Time (32)
	Optimism (44)	Partner (35)	Control (18)	Meditation (26)	Body (30)
	Strength (40)	Spouse (26)	Knowing (15)	Relaxation (26)	Need (22)
	Day (37)	Physical (23)	Patience (15)	Mind (25)	Sun (19)
	Mental (31)	People (21)	Rheumatologist (15)	Nutrition (23)	Break (12)
Associated variable category, test value and p value					
	Age (41–60 years), 7.84, p<0.001 Age (61–87 years), 3.24, p=0.002	Age (18–40 years), 5.45, p<0.001	Age (61–87 years), 2.43, p=0.018	Age (41–60 years), 5.13, p<0.001	
	Years from diagnosis (11–70 years), 9.43, p<0.001	Years from diagnosis (0–10), 3.30, p=0.001			
	Education (up to secondary), 8.52, p<0.001			Education (higher), 2.27, p=0.026	
	Pain/discomfort (high), 8.84, p<0.001			Pain/discomfort (low), 4.71, p<0.001	
		Kidneys (involvement), 2.40, p=0.019	Kidneys (involvement), 2.18, p=0.032	Kidneys (no involvement), 2.77, p=0.007	
		Bloodstream (involvement), 2.42, p=0.018		Bloodstream (no involvement), 4.51, p<0.001	
	Central nervous system (no involvement), 7.49, p<0.001			Central nervous system (no involvement), 3.69, p<0.001	
	Lungs (no involvement), 7.87, p<0.001			Lungs (no involvement), 2.63, p=0.011	
	Gender (female), 8.26, p<0.001			Functional autonomy (low), 4.81, p<0.001	
				Anxiety/depression (low), 4.83, p<0.001	
				Employment (part-time), 4.41, p<0.001	
				Antiphospholipid syndrome (no), 4.14, p<0.001	
				Disease (under control), 3.53, p=0.001	
				Heart (no involvement), 3.47, p=0.001	
				Joints (involvement), 2.35, p=0.022	
				Muscles (no involvement), 2.17, p=0.032	
				Skin (no involvement), 2.01, p=0.046	

Cluster 1 ‘positive attitude’ is described by keywords that emphasise the *mental positive attitude* that the respondents have, *living* with *optimism* and taking their *strength* from the presence of their *children*. This coping strategy is more frequent in patients more than 40 years of age with no critical organ involvement (such as the lungs or the central nervous system).

Cluster 2 ‘social support’ has to do with the *support*, *love* and *understanding* that the respondents receive from many *people*, including their *partner* (*husband*, *spouse*), *family* and *friends*. This coping strategy appears associated with age 18–40 years with kidney involvement and bloodstream involvement.

Cluster 3 ‘trust in medical treatments’ is described by words related to *good medical treatments*, including *drugs*, *controls* and *follow-ups* with the *rheumatologist*. This is more frequent in patients aged 61 years or more with kidney involvement.

Cluster 4 ‘healthy habits’ has to do with a number of *active* behaviours for staying *healthy*, such as *working*, doing *sports* and *exercises*, *meditation* and *relaxation*, and paying attention to *nutrition* (*eating*, *diet*). This cluster is associated with joint involvement and low functional autonomy, but with the disease under control, lower levels of anxiety/depression and lower pain/discomfort, and absence of many other organ involvements (bloodstream, antiphospholipid syndrome, central nervous system, heart, lungs, muscles and skin).

Finally, cluster 5 ‘to avoid stress’ includes a number of words related to *avoiding stress* (*pace*, *yoga*, *sleep*, *rest*, *break*), *regular medications* (eg, *hydroxychloroquine*) intake and a potentially stressful situation like *sun* exposure. This cluster does not appear significantly associated with the variables under study, meaning that these words were equally (ie, not differentially) used by participants with different characteristics.

## Discussion

Our work focused on the strategies that patients with SLE report when they describe how they face their condition.

In this paper we were able to identify, in a large sample of patients with SLE, the most frequent words used by patients to describe their coping strategies, grouping them into significant themes. Five themes, corresponding to as many coping strategies, were identified: positive attitude, social support, trust in medical treatments, healthy habits and avoid stress. All these strategies were quite important for patients, ranging from 10.77% (medical treatments) to 25.46% (social support) of the total word occurrences.

The strategies appeared both emotion-focused (like positive attitude) and problem-focused (like trust in medical treatments), and both approach (seeking social support) and avoidance (like avoiding stress), covering several classic coping classifications.^24 26^ Moreover, some of them clearly corresponded to the coping strategies that were recently identified in qualitative studies with patients with SLE.^25^

Interestingly, in this paper, we were able to test the possible association between the themes and some patient characteristics. For example, it appeared quite clear that words related to keeping a ‘positive attitude’, despite the high level of pain/discomfort, were frequently reported by older patients (aged 41–60 and 61–87). On the other hand, ‘seeking social support’ was more typical of younger patients (aged 18–40), with less years from diagnosis and with some signs of organ involvement (bloodstream and kidney involvement). The importance of ‘trust in medical treatment’ was reported by the oldest participants (aged 61–87) with kidney involvement. Finally, a coping strategy based on active behaviours for staying healthy (like nutrition, movement and work) was typical of patients who, despite their low functional autonomy, reported less severe lupus, under control and with joint involvement only (see the ‘healthy habits’ cluster).

These results have strong implications for clinical practice and for interventions aimed at improving patients’ quality of life. First, describing the content of the coping strategies, the study provides a detailed picture of the more frequently used strategies by a large sample of patients. Second, our study shows that patients with certain characteristics (both demographics and disease-related) may be willing to use some specific coping strategies. These results are particularly important if we consider that learning to adapt to a lifetime of having lupus may require replacing old coping methods with more effective approaches and that identifying successful coping strategies may be an important goal of both individual and group interventions.^32^

Some limitations of the present study should be recognised. The first limitation has to do with the selection bias of participants. Indeed, the survey ‘Living with Lupus in 2020’ was distributed through patient organisations and the respondents might not be statistically representative of the entire population of patients with SLE. For example, the sample was unbalanced in some patient characteristics (ie, gender). Nevertheless, the association between the thematic clusters and patient characteristics (ie, illustrative variables) was found in a large sample, suggesting interesting hypotheses that could be tested with a more representative sample.

A second limitation is represented by the short length of the statements describing coping strategies (around 10 words, which were subsequently translated to English for the analyses). With more extensive descriptions, the number of words and the lexicon could be higher. However, the shortness of the answers was functional to keep the questionnaire short and to obtain answers of comparable length. Moreover, following the T-LAB logic,^28^ the low number of words used by each participant did not represent a real obstacle for data analysis and interpretation, if we consider the very high number of cases, which produced a large and rich data set.

Third, the method of assessing patient characteristics was based on self-report items, rather than multi-item validated scales. This could limit the construct validity and the possibility of differentiating between different psychological symptoms (eg, anxiety and depression) as well as quality of life dimensions.

A final remark should be done about the possible interpretations of causality in the results. From our data, it is not possible to identify the more adaptive coping strategies for patients with SLE. For example, the fact that one strategy is statistically associated with low organ involvement and with a higher autonomy does not allow to drive conclusions about the causal direction (eg, is it because of this coping strategy that the level of autonomy is higher or is it that patients with a higher level of autonomy are more willing to adopt this coping strategy?). Further longitudinal studies with patients with SLE are necessary to clarify this aspect.

## Data Availability

Data are available upon reasonable request.
