# Supplementary material for: Coping with systemic lupus erythematosus in patients’ words
Source: Lupus Sci Med. 2022 May 12;9(1):e000656. doi: 10.1136/lupus-2022-000656 (PMC9109093; doi:10.1136/lupus-2022-000656)
Supplement: Supplementary data [file lupus-2022-000656supp001.pdf]

Supplementary materials

Table 1. Clusters with the complete list of key-words.

	Cluster 1 "positive attitude"	Cluster 2 "social support"	Cluster 3 "trust in medical treatments"	Cluster 4 "healthy habits"	Cluster 5 "to avoid stress"
Total occurrences	1434 (22.58%)	1617 (25.46%)	684 (10.77%)	1317 (20.74%)	1299(20.45%)
Words (occurrences)	positive (163)	family (445)	good (105)	exercise (120)	rest (287)
	child (124)	support (192)	treatment (55)	work (88)	medication (252)
	life (94)	friend (119)	medical (43)	sport (65)	sleep (114)
	think (89)	husband (96)	faith (34)	walk (62)	stress (111)
	attitude (73)	medicine (94)	care (34)	diet (48)	regular (47)
	pain (58)	love (73)	drug (32)	dog (34)	pace (47)
	daughter (47)	doctor (63)	listen (27)	healthy (34)	hydroxychloroquine (46)
	help (45)	activity (57)	therapy (25)	movement (31)	yoga (35)
	disease (45)	understanding (47)	know (22)	eat (29)	avoid (33)
	live (44)	positivity (42)	follow-up (19)	active (27)	time (32)
	optimism (44)	partner (35)	control (18)	meditation (26)	body (30)
	strength (40)	spouse (26)	knowing (15)	relaxation (26)	need (22)
	day (37)	physical (23)	patience (15)	mind (25)	sun (19)
	mental (31)	people (21)	rheumatologist (15)	nutrition (23)	break (12)
	son (29)	contact (20)	specialist (13)	balance (23)	able_to (12)
	stay (27)	social (20)	symptom (11)	food (22)	management (12)
	trying (26)	nature (18)	plan (11)	working (22)	reduce (11)
	accept (20)	relationship (15)	limit (11)	calm (18)	little (10)
	willpower (18)	supportive (14)	God (10)	painkiller (18)	tranquility (10)
	moving (18)	lupus (14)	condition (10)	job (17)	sufficient (9)
	bad (17)	close (12)	knowledge (9)	training (16)	relax (9)
	character (17)	psychological (11)	situation (9)	peace (15)	regularly (8)
	desire (17)	hobby (11)	Trust (9)	physiotherapy (14)	tablet (8)
	grandchild (13)	daily (10)	team (8)	meds (14)	steroid (8)
	try (13)	emotional (9)	learning (7)	routine (14)	oil (7)
	positively (12)	group (8)	illness (7)	quiet (13)	acupuncture (7)
	acceptance (12)	happy (8)	moment (7)	swim (13)	adequate (7)
	mood (11)	music (8)	monitor (7)	home (13)	check (6)
	understand (11)	horse (7)	morale (7)	cortisone (13)	managing (6)
	normal (10)	financial (7)	diagnosis (7)	entourage (13)	emotion (6)
	fight (10)	meaningful (6)	alternative (6)	environment (13)	mindfulness (6)
	hope (10)	parent (6)	calmly (5)	cycle (12)	flexible (6)
	living (9)	talk (6)	cannabis (5)	kid (12)	sunscreen 6()
	stubbornness (9)	relaxing (5)	distraction (5)	manage (12)	pressure (5)
	move (8)	reading (5)	fortitude (5)	lot (11)	schedule (5)

	personal (8)	network (5)	hospital (5)	strong (11)	energy (5)
	humor (8)	optimistic (5)	psychotherapy (5)	relief (10)	low (5)
	courage (8)	loving (5)	recognize (5)	joy(10)	avoidance (5)
	enjoy (7)	information (5)	visit (5)	lifestyle(10)	blood (5)
	look (7)	internist (4)	year (4)	dance(9)	drink (4)
	give_up (7)	helping (4)	surround (4)	continue(8)	CBD (4)
	professional (7)	fun (4)	access (4)	anti-inflammatory (8)	finding(4)
	remission (7)	association (4)	feel (4)	right (8)	light(4)
	stability (7)	mentally (4)	correct (4)	play (8)	start(4)
	thought (7)	moral (4)	corticosteroids (4)	vitamin (8)	plenty(4)
	state (6)	patient (4)	confidence (4)	serenity (7)	task(4)
	problem (6)	peer (4)	pray (4)	awareness (7)	
	perseverance (6)	security (4)	periodic (4)	bath (7)	
	forward (6)	travel (4)		change (7)	
	adapt (6)	stable (4)		inner (7)	
	affection (6)			holiday (7)	
	believe (6)			holidays (6)	
	everyday (5)			heat (6)	
	feeling (5)			hour (6)	
	mindset (5)			pilates (6)	
	practice (5)			animal (6)	
	self-control (5)			determination (6)	
	pass (5)			bike (6)	
	spirits (5)			breathe (6)	
	worry (5)			busy (6)	
	mother (4)			focus (6)	
	motivation (4)			supplement (6)	
	sick (4)			prednisolone (6)	
	psychologist (4)			water (6)	
	important (4)			week (5)	
	cope (4)			positivism (5)	
	career (4)			stress-free (5)	
				currently (5)	
				hot (4)	
				laughter (4)	
				man (4)	
				outdoor (4)	
				month (4)	
				cure (4)	
				companion (4)	
				effort (4)	
				discipline (4)	
				garden (4)	
				great (4)	
				gym (4)	
				force (4)	
				stretch (4)	
				sea (4)	
				study (4)	
				recreation (4)	
				regulate (4)	
				resilience (4)	
